# A discrete choice experiment of preferences for genetic counselling among Jewish women seeking cancer genetics services

**DOI:** 10.1038/sj.bjc.6603451

**Published:** 2006-11-14

**Authors:** S Peacock, C Apicella, L Andrews, K Tucker, A Bankier, M B Daly, J L Hopper

**Affiliations:** 1 British Columbia Cancer Agency, Cancer Control Research, 675 West 10th Avenue, Vancouver, British Columbia V5Z 1L3, Canada; 2 Department of Health Care and Epidemiology, University of British Columbia, 5804 Fairview Avenue, Vancouver, British Columbia V6T 1Z3, Canada; 3 Centre for Molecular, Environmental, Genetic and Analytic Epidemiology, School of Population Health, The University of Melbourne, Level 2, 723 Swanston Street, Carlton, Victoria 3053, Australia; 4 Hereditary Cancer Clinic, Prince of Wales Hospital, High St, Randwick, New South Wales 2031, Australia; 5 Genetic Health Services Victoria, Royal Children's Hospital, Flemington Road, Parkville, Victoria 3052, Australia; 6 Fox Chase Cancer Center, 333 Cottman Avenue, Philadelphia, PA 19111-2497, USA

**Keywords:** Ashkenazi, breast cancer, BRCA1 and BRCA2, discrete choice experiment, client preferences, genetic counselling

## Abstract

To determine which aspects of breast cancer genetic counselling are important to Ashkenazi Jewish women, a discrete choice experiment was conducted. Participants consisted of 339 Australian Ashkenazi Jewish women who provided a blood sample for research used to test for Ashkenazi Jewish ancestral mutations in the genes BRCA1 and BRCA2, and were offered their genetic test result through a cancer genetics service. Main outcome measures were women's preferences for, and trade-offs between, the genetic counselling aspects of providing cancer, gene, and risk information (information); giving advice about cancer surveillance (surveillance); preparing for genetic testing (preparation); and, assistance with decision-making (direction). Respondents most valued information, about twice as much as advice about surveillance, four times as much as preparation for testing, and nine times as much as assistance with decision-making, which was least valued. Women's preferences were consistent with the major goals of genetic counselling, which include providing information and surveillance advice, and avoiding direction by facilitating autonomous decision-making. There were differences between the women in which aspects they most favoured, suggesting that counselling that elicits and responds to clients’ preferences is more likely to meet clients’ needs.

Shortly after the cloning of the breast cancer susceptibility genes BRCA1 and BRCA2, three specific mutations in BRCA1 and BRCA2 were found to be 20 times more common in individuals of Ashkenazi Jewish descent than in the general population (Struewing *et al*, 1997). These ancestral mutations are thought to account for 20% of all breast cancer in Ashkenazi Jewish women (Satagopan *et al*, 2001). Carriers of a mutation in either of these genes are at 40–60% lifetime risk of developing breast cancer and 20–40% lifetime risk of developing ovarian cancer (Robson *et al*, 1997; Fodor *et al*, 1998; Hartge *et al*, 1999; Satagopan *et al*, 2001).

In the past decade, there has been a rapid increase in the demand for, and availability of, breast cancer genetics services (Thompson *et al*, 1995). These services aim to provide women with an estimate of their risk of developing breast and ovarian cancer based on their family history, and in some cases, genetic testing for mutations in BRCA1 and BRCA2 is offered. These services may further aim to improve psycho-social outcomes for clients by inclusion of other specialities and techniques, including, facilitating autonomous decision-making about genetic testing and risk-reduction options, preparing clients for the possible outcomes of genetic testing, and offering family or group therapy sessions. In recent years, many services have established multidisciplinary teams including surgeons, oncologists, and gynaecologists so that breast and ovarian cancer surveillance advice can be provided.

Outcomes of genetic counselling may relate to *process issues*, or may relate to desired *client outcomes*. Process issues, such as satisfaction with consultation waiting time, location, duration of counselling sessions, and type of service provider, have been examined in a number of studies (Shiloh *et al*, 1990; Bernhardt *et al*, 2000; Brain *et al*, 2000; Metcalfe *et al*, 2000; Wilson *et al*, 2000; Holloway *et al*, 2004; Pieterse *et al*, 2005a, 2005b). Client outcomes are more difficult to measure than process issues, but are more salient for evaluating and informing service delivery (Clarke *et al*, 1996; Cappelli *et al*, 2001). Desired client outcomes, such as reduced anxiety, improved genetic knowledge, and risk perception, have received some attention in the literature (Bernhardt *et al*, 2000, Metcalfe *et al*, 2000; Pieterse *et al*, 2005a, 2005b).

Desired client outcomes may be influenced by several factors including clients’ needs, their expectations and their preferences before counselling, and also the fulfilment, or perceived fulfilment, of these factors after counselling. Studies of expectations before genetic counselling have shown that clients often do not know the procedure or structure of the counselling appointment, sometimes resulting in the client feeling that he or she was inadequately prepared (Hallowell *et al*, 1997; Stadler *et al*, 1998; Berkenstadt *et al*, 1999; Bernhardt *et al*, 2000; Holloway *et al* 2004). Studies of preferences measured before genetic counselling have further shown that clients have high preference for information, although it is difficult to interpret findings regarding preferences for aspects other than information given clients’ lack of knowledge about the counselling process before attendance (Cohn *et al*, 2003; Tiller *et al*, 2005).

In this study, we elicited preferences for genetic service delivery in Jewish women who had previously participated in a BRCA1 and BRCA2 genetic testing programme, and so had knowledge of the procedures of genetic counselling. The aim of this study was to measure strength of preference for the different aspects of genetic counselling outcomes in these women. Discrete choice experiments are a theoretically valid technique developed by psychologists and economists for eliciting preferences, and are particularly well suited to eliciting preferences for the wide range of health and nonhealth benefits potentially derived from using health services (Ryan, 1999, 2004; Ryan and Farrar, 2000; Sculpher *et al*, 2004). We used a discrete choice experiment to provide robust information about what aspects clients *want most* from genetic counselling, the *relative value* they place on different counselling aspects, and *how much value* they place on the different aspects.

## MATERIALS AND METHODS

### Study sample

Eligible women were participants in the Australian Jewish Breast Cancer Study (AJBCS) (Apicella *et al*, 2003) who had previously received their genetic test results and enrolled in the study at least 6 months earlier. Participants recruited in the previous 6 months were excluded because many had not yet attended a genetic clinic for genetic counselling and result disclosure appointments. At recruitment, participants were administered a structured questionnaire developed by the Breast Cancer Family Registry, provided a blood sample that was tested for the Jewish ancestral mutations in BRCA1 and BRCA2, and received genetic information (John *et al*, 2004). Most women subsequently received their genetic test result through one of six state-funded cancer genetics services, as described previously (Apicella *et al*, 2006).

### Discrete choice experiment

A follow-up questionnaire was mailed to all 339 participants. The questionnaire included a discrete choice experiment for preferences of attributes (aspects) of genetic counselling. These attributes were identified through reviews of the peer reviewed and policy/practice literature on genetic counselling, consultation with clinical geneticists, genetic counsellors, heads of genetic services, psychologists, and interviews with participants (Apicella *et al*, 2006). The four identified attributes included in the discrete choice experiment were: providing and explaining cancer, gene and risk information (Information); explaining options and giving advice about appropriate surveillance for breast and ovarian cancer (Surveillance); preparing for the outcomes of genetic testing (Preparation); and, receiving help in deciding whether or not to have a genetic test (Direction).

**Figure 1:**
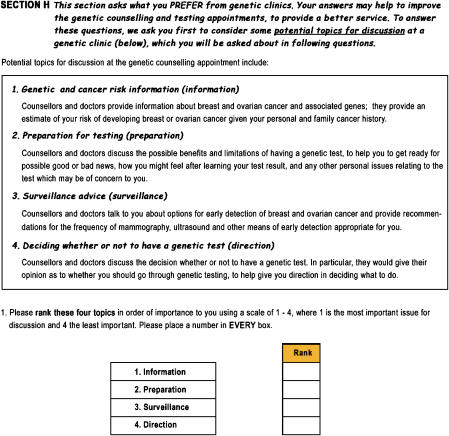
Attribute-ranking question used in the follow-up questionnaire.

Descriptions were developed for each of the counselling attributes to help participants understand the nature of each that they were being asked to consider (see Figure 1). The critical task in describing attributes was to ensure they captured the desired client outcomes relevant to women attending the participating genetics services. To this end, clinical geneticists and counsellors who counselled the participants at these services played a central role in developing the descriptions. The four items and their descriptions were developed to capture information, emotional and psychosocial support, surveillance advice, and direction outcomes relevant to their practices.

**Figure 2:**
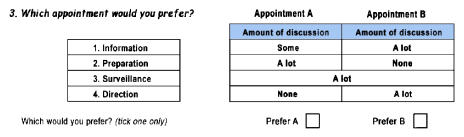
Example of a discrete choice experiment question used in the follow-up questionnaire.

Respondents were asked to rank their preferences for each of the four attributes at a genetic counselling appointment, where rank one is their most preferred and rank four is their least preferred. Strength of preference for each was then measured using a discrete choice experiment (see Figure 2).

Discrete choice experiments measure preferences by analysing individuals' responses to questions about choices they would make in hypothetical, yet realistic, situations. Respondents are asked to choose their preferred option (in this case different counselling appointments described by a unique combination of different levels for each of the counselling attributes) from a series of pairwise choices. A probabilistic discrete choice model was used to analyse the data.

Each discrete choice question asked respondents to choose between two hypothetical genetic counselling appointments described in terms of different levels of the four counselling attributes. Scenarios were paired randomly. Three attribute levels were used in the experimental design according to the amount of discussion – none, some, or a lot – devoted to each attribute during a counselling appointment. Respondents were therefore asked which of the two hypothetical genetic counselling appointments they would prefer, where alternative appointments were described in terms of different levels for the four attributes (see Figure 2). The SPEED computer package was used to select the optimal subset of scenarios, making the number of discrete choice questions manageable (Bradley, 1991).

An internal consistency test was included with the discrete choice experiment. This involved presenting a choice of counselling appointments with attribute levels such that all respondents should choose the same appointment. Only responses that showed internal consistency were included in the subset for analysis.

### Statistical analysis

A random effects probit regression model was used to analyse the discrete choice data (Ryan and Farrar, 2000) using Stata v8.2.

## RESULTS

### Respondents

Of the 339 AJBCS participants who were sent the follow-up questionnaire (average 44 months after enrolment in the study), 256 (76%) were completed and returned. Table 1 shows the mean (s.d.) age of respondents was 52.6 years (12.0). The mean number of children was 2.0 (1.2). The mean genetic knowledge score was 6.8 (1.9). The mean State Trait Anxiety Inventory score was 38.8 (10.4). Thirty participants were found to be mutation carriers. Older women had more children (*P*<0.01) and were more likely to have had cancer (*P*<0.01). Younger women were more likely to have a university degree (*P*<0.01), and had better genetic knowledge (*P*<0.01). Recent death in the family was associated with a strong family history of cancer (three or more cases of breast or ovarian cancer in first- and second-degree relatives) (*P*<0.01) and recent cancer diagnosis in the family (*P*<0.01).

### Simple attribute ranking

Simple attribute-ranking preferences are presented elsewhere (Apicella *et al*, 2006). More than 60% of respondents ranked information as the most important attribute. Approximately 20 and 15% of respondents ranked surveillance and preparation as the most important attribute, respectively. Only 5% ranked direction as most important. More than 50% of respondents ranked direction as the least important attribute. Approximately 20% ranked surveillance, 25% preparation, and 5% information as least important, respectively. Preferences were not significantly different between the mutation carriers and noncarriers (Apicella *et al*, 2006). Rankings for the most important attribute for mutation carriers and noncarriers, respectively, were 67 and 61% for information; 13 and 20% for surveillance; 17 and 14% for preparation; 3 and 5% for direction.

### Discrete choice experiment

Of the 209 women who completed all five discrete choice questions, 193 (92%) passed the consistency test. The random effects probit model was fitted using responses from these 193 respondents (Table 2). All main effects, the *β*_j_ coefficients, were positive and significant at the 1% level, except direction, which was significant at the 5% level, showing that increased amounts of discussion for each attribute were associated with increased utility (attributes are monotonically increasing in levels).

The most important attribute was information, followed by surveillance, and then preparation (Table 2). The least important attribute was direction. The main effects, statistically significant for all four attributes, showed that increasing the level (from none to some or from some to a lot) of discussion of information, utility increased by 0.997. Increasing the level of discussion of surveillance, preparation, and direction increased utility by 0.463, 0.267, and 0.114, respectively.

Table 3 shows the relative value, or importance, of the different attributes, given by the ratios (*β*_info_/*β*_dirn_) of the main effects. In this study, women would give up 8.75 units of discussion of direction to get an extra unit of discussion of information. That is, information was found to be almost nine times as important as direction. Alternatively, it could be viewed that women would only give up 0.11 units of discussion of information to get an extra unit of discussion of direction. These data also show that discussion of information was more than three times as important as discussion of preparation, and more than twice as important as discussion of surveillance. Similarly, discussion of surveillance was four times more important than discussion of direction, and discussion of preparation was more than twice as important as discussion of direction.

The effects of respondents’ personal characteristics and experiences on the relative value of attributes were examined to determine whether preferences vary systematically between respondents. This was performed by modelling potential interactions between characteristics and preferences in the regression model (segmentation analysis). Personal characteristics included demography, education, parity, psychological well-being, personal and family cancer history, mutation carrier status, and genetic knowledge. No statistically significant relationships were identified.

## DISCUSSION

Women had highest preference for information, valuing it almost nine times more than direction in decision-making about genetic testing. This is consistent with the major aims of cancer genetic services, which include providing cancer, genetic and risk information, and facilitating autonomous decision-making. Women also had high preference for discussion of breast and ovarian cancer surveillance options, valuing it twice as much as preparation for possible outcomes of genetic testing, and four times as much as direction in decision-making about genetic testing. This provides evidence that the shift towards a multidisciplinary team, which includes oncologists and surgeons able to provide surveillance advice is consistent with women's preferences for genetic counselling. The discrete choice experiment results are entirely consistent with results from attribute-ranking questions presented in Apicella *et al* (2006).

Although many women preferred information most, and assistance with decision-making least, there were differences between women in which aspects they most favoured. In particular, we found that some women valued preparation highly, whereas others placed least value on this aspect of genetic counselling. Since analysing these data, we have become aware of another study which has similarly found that in general, clients preferred information most and emotional support least, although a subset had high preference for emotional support (Pieterse *et al*, 2005b).

The results from this study suggest that a uniform and structured genetic counselling appointment may not be the best method of service delivery, as preferences vary between clients, and fulfilment of clients’ preferences are important for achieving desired client outcomes. This has also been demonstrated recently by Pieterse *et al* (2005b), who showed that desired client outcomes such as increased perceived personal control and reduced anxiety are significantly positively associated with clients’ perceptions that their preferences for service delivery were met. That is, studies that help to identify client preferences both in general and in specific subgroups may assist in improving the delivery of genetic services.

Participants of this study have undergone genetic counselling, and most have received their genetic test result, meaning that they are well placed to identify attributes of genetic services that are important to users of those services (Genetic Interest Group, 1998; Royal College of Physicians, 1998). The importance of eliciting preferences from respondents with first-hand experience of genetics and genetics services in understanding the attributes of genetics services has been recognised elsewhere (Wilson *et al*, 2000).

Given *a priori* expectations, the discrete choice experiment results are plausible, and provide further evidence that the technique can be successfully applied in health care. Discrete choice experiments have been shown to provide internally valid and consistent responses (Viney *et al*, 2002; Ryan and Gerard, 2003; Ryan *et al*, 2003). However, this may depend on study context (Ryan and Gerard, 2002), and there is some evidence that some respondents may not trade-off attributes, but adopt simpler decision heuristics (Scott, 2002; Lloyd, 2003). Although the choices presented to respondents are hypothetical, this allows researchers to have complete control over experimental design and ensures statistical robustness (Ubach *et al*, 2003). An important question with any stated preference technique is that of external validity: would respondents make the same choices in reality? Surveying respondents with first-hand experience genetics services, and evidence from other areas such as the valuation of environmental goods and services means that we can be optimistic (Wilson *et al*, 2000; Ryan, 2004). Clearly, future research on these topics is warranted.

It remains to be seen whether results from this study are generalisable to other populations and to other types of genetic testing, owing to differences in the characteristics of participants of this study and of other populations seeking cancer genetic counselling. Such differences include; specific testing with a more definitive outcome was conducted in the Ashkenazi Jewish population, whereas there is often no definitive outcome from BRCA1 and BRCA2 testing in the general population; the extent of family cancer history of participants may differ from other populations undergoing BRCA1 and BRCA2 testing; and, other cultural groups may value counselling outcomes differently. Nonetheless, a study of preferences for cancer genetic counselling in a Dutch population found that clients had similar preferences to those identified in this study of Australian Jewish women (Pieterse *et al*, 2005b), suggesting that findings from this study may apply to other populations.

The discrete choice experiment technique could be further used to elicit client preferences for a range of other health and nonhealth objectives relating to genetics services. In particular, access to genetics services may be improved if genetics testing becomes available through general practitioners on a widespread basis. However, this might be associated with a more limited service for clients, as most general practitioners will not have the time or level of genetics training as found in dedicated genetic clinics. A discrete choice experiment could be used to measure the value that clients place on improving access to genetics services relative to the amount of information and other aspects of genetic counselling that they would receive from their local family doctor compared to a genetic clinic.

## Figures and Tables

**Table 1:** Descriptive characteristics of respondents

**Personal characteristic**	***n***	**Per cent**
*Age* N=*210 (years)*		
25–54	127	(60)
55–74	74	(35)
75+	9	(4)
Mean (s.d.)	52.56 (11.96)	
		
*Number of children* N=*205*		
0	33	(16)
1	21	(10)
2 or 3	138	(67)
4 or more	13	(6)
Mean (s.d.)	1.97 (1.19)	
		
*University degree* N=*210*		
Yes	104	(50)
No	106	(50)
		
*Breast cancer* N=*208*		
Yes	72	(35)
No	136	(65)
		
*Family history (first- and second-degree relative)* N=*207*		
1 case breast cancer	74	(36)
2 cases breast cancer	51	(25)
3 or more cases breast cancer	52	(25)
Carrier of ancestral mutation	30	(14)
		
*Genetic test result received* N=*210*		
Yes	187	(89)
No	23	(11)
		
*Genetic knowledge (max. 9)* N=*209*		
1–3 (poor)	19	(9)
4–6 (fair)	44	(21)
7–9 (good)	146	(70)
Mean (Std. dev)	6.75 (1.94)	
		
*Anxiety (STAI-Trait)* N=*206*		
<40	118	(57)
40–54	67	(33)
55–70	21	(10)
Mean (s.d.)	38.84 (10.43)	
		
*Recent death of a relative* N=*202*		
Yes	44	(22)
No	158	(78)
		
*Recent cancer of a relative* N=*202*		
Yes	42	(21)
No	160	(79)

**Table 2:** Random effects probit model results

	**Main effect^a^ (*β*_j_)**	**s.e.**	**95% Cl**	***P*>∣*z*∣**
Information	0.997	0.113	0.776–1.218	0.000
Surveillance	0.463	0.036	0.393–0.533	0.000
Preparation	0.267	0.045	0.182–0.353	0.000
Direction	0.114	0.047	0.023–0.206	0.014

CI=confidence interval.

Individuals=193; observations=965.

Log-likelihood=−510.209; Wald *χ*^2^(4)=250.15; Prob.>*χ*^2^=0.000.

a An alternative approach to estimating the main effects model is to use dummy variables for categorical attribute levels in the independent variable set. However, this approach becomes problematic owing to colinearity in the set of dummy variables.

**Table 3:** Relative value of attributes: ratios of main effects

	**Main effect (*β*_j_)**	***β*_j_/*β*_info_**	***β*_j_/*β*_surv_**	***β*_j_/*β*_prep_**	***β*_j_/*β*_dirn_**
Information	0.997	1.00	2.15	3.73	8.75
Surveillance	0.463	0.46	1.00	1.73	4.06
Preparation	0.267	0.27	0.58	1.00	2.34
Direction	0.114	0.11	0.25	0.43	1.00
